# Teilhard de Chardin’s oeuvre within an ongoing discussion of a gene drive release for public health reasons

**DOI:** 10.1186/s40504-017-0064-8

**Published:** 2017-12-20

**Authors:** Anto Čartolovni

**Affiliations:** Catholic University of Croatia, Ilica 242, 10000 Zagreb, Croatia

**Keywords:** CRISPR/Cas9, Gene drives, Climate change, Vector-borne disease, Pierre Teilhard de Chardin, Public health, Ethics

## Abstract

Within the domain of public health, vector-borne diseases are among the most vehemently discussed issues. Recent scientific breakthroughs in genome editing technology provided a solution to this issue in the form of a gene drive that might decrease and even eradicate vector-borne diseases. Gene drives are engineered, and designed genes that can break typical inheritance rules and be passed to almost all of the carrier’s offspring. This genome editing and gene drive technology has become a powerful tool for ecological and environmental engineering, through which man can manipulate his surroundings, adjusting it to himself and directly mastering evolution and the ecosystem. Although the gene drive technology has been perceived as promising in the public health domain, ecological implications of its use are not to be underestimated. The primary aim of this paper is to overcome the ongoing discussion which mostly focuses on whether priority should be given to the environment or to public health, and to find an adequate answer and solution. In this quest to find the proper answer and solution, Pierre Teilhard de Chardin’s thought might be useful, especially his concepts of the biosphere and the noosphere which may provide some clarifications as to why we are at the moment so cautious with gene drive technology and how we need to *move towards* a better common future on earth.

## Introduction

Albeit the actual existing polarisation among the positions for or against the releasing of gene-drive modified organisms’ (farther on GDMO) into the environment, I will argue here that the legacy of the French philosopher and scientist Pierre Teilhard de Chardin may offer a suitable approach to the ongoing discussion. Humanity has become aware of big global changes, and its role regarding these changes, we now see ourselves as a strong geophysical force operating side by side with natural forces. Moreover, the planet and the environment do not stand anymore in the self-governing Holocene period, but instead, as defined by (Crutzen & Stroemer, 2000), in a new Anthropocene epoch^1^ (Crutzen & Stroemer, 2000). There is much evidence to describe the shift and arrival of this new epoch, ranging from atmospheric, hydrospheric, biospheric, and geospheric processes profoundly altered by humans (Keulartz & Bovenkark, 2016). Nevertheless, although a similar meaning of the concept was proposed in 1873 by the Italian geologist Antonio Stoppani, in the form of the precedent concept of an “Anthropozoic era,” Crutzen and Stroemer propose the industrial era as the starting point for the proposed Anthropocene epoch (Keulartz & Bovenkark 2016). One of the most controversial twist periods in the Anthropocene for both society and the environment was the accelerating drive, dated from the middle of the last century where humanity made enormous progress not only in understanding the molecular and genetic bases of life, but also in synthesising life itself (Steffen et al. 2011). No doubt that climate change, which we are experiencing now, has significantly contributed to the quick spread and dissemination of insect vector-borne diseases^2^ as one of the largest problems within the public health domain. Recently society has sought to confront itself with these vector-borne diseases, broadly, across the globe, and several approaches have been applied to fight these diseases; some with more success than others (Macer 2005). Even the significant trails of the Anthropocene and climate change have contributed to the empowerment of the evolutionary organism resistance to already used control methods.

The main vectors for the transmission of diseases are *Anopheles* mosquitoes for malaria, especially *Anopheles gambiae*, and *Aedes aegypti* for dengue virus. Furthermore, out of 460 different *Anopheles species,* 30 to 40 are vectors for the *Plasmodium* parasite (Kamaraddine 2012). Recent statistical data of the WHO Malaria report shows that there has been a globally significant decline in malaria cases, 18%, since 2000, as well as a 48% decline in the number of deaths. In 2015 438,000 people died from malaria (WHO 2015), with most of the cases being from Africa; in 2015, for the first time, there were no reported cases of malaria in Europe. This statistical decline demonstrates that control and fighting methods have been successful, but are still not enough, taking into consideration the half a million deaths in 2015 alone. The complicated situation with the problems of vector-borne diseases has motivated scientists to find new solutions in the fight against them. A significant contribution to finding new control methods came from recent gene editing tools, which opened up a new and paradigmatic page in life and science(Macias & James 2015).

The discoveries of new gene editing tools such as Clustered Regularly Interspaced Short Palindromic Repeats (CRISPR) (Charpentier & Doudna, 2013) enabled scientists to effectively create a decades-old idea of a “selfish gene” (Burt 2003), which through breeding processes enabled the spread of particular genetic alterations through entire species. Spreading the genome alterations throughout the population opens up the possibility for scientists to isolate the gene responsible for the transmission of the disease or to inhibit the species from reproducing. We must recognise that this new approach would not only have implications for human health and the vectors, but also for the environment and the entire ecosystem that would receive characteristics of ecological or environmental engineering (Esvelt et al. 2014).

The evaluation of these changes does not need to be looked at through an anthropocentric lens, where only outcomes that are important for humans would be perceived, but the changes should be evaluated through the lens of the ecosystem and biotic community (NAS 2016). However, focusing only on the biotic community^3^ might lead to another kind of extreme in the form of biocentrism, where actual human benefits are underestimated. Taking a position of either the former or the latter perspective might lead to irresponsible conduct and severe decisions. Therefore, within this paper we will take an approach based on Pierre Teilhard de Chardin’s legacy where the possibility of GDMO release into the environment is not apriori excluded, nor any possible and foreseeable ecological implications. Using an approach based on Teilhard’s oeuvre we try to shed light on the ongoing discussion in the form of a diagnostic of a present that may provide useful moral deliberation involving “an anamnesis of the past and a prognostic of the future” (Zwart et al. 2016a, p. 2). Furthermore, Teilhard’s oeuvre corresponds perfectly to the present Anthropocene epoch, recently recognised and appraised by Dr. Hub Zwart as a “philosopher of the Anthropocene *par excellence*” (Zwart, 2016b, p. 398). In fact, applying Teilhard’s oeuvre to the GDMO discussion we try to transcend the actual consequentialist concerns and offer a value-based approach integrating not only our interests, but also the interests of every being belonging to the biotic community. Not only should the uncertainty and unknowns over the science of the gene drive technology be in a primary focus, but also the ecological implications that might occur in cases where we can foresee the implications of a GDMO release. Therefore, this approach differs in some way from those existing within GDMO discussion; however, it has some similarities of common shared values with the One Health (OH) approach that has already been applied within public health. (Capps et al. 2015). Even though the shared values such as ecosystem and biodiversity are perhaps in common, Teilhard’s concept puts the emphasis on the biosphere’s stability, and not underestimate its potential plasticity. Furthermore, the biosphere does not represent something intangible that needs to infringe every action that leads to its modification, but possesses a certain capacity of adjustment to the changes performed similar to plasticity. Moreover, to understand its stability and plasticity we need to acquire more interdisciplinary knowledge that results from De Chardin’s noosphere (Teilhard de Chardin, 1999).

The first aim of this paper would be to propose Teilhard de Chardin’s conceptual difference between biosphere and noosphere that might provide some clarifications when asking whether to introduce the GDMOs or not and, if yes, when. The second aim is to investigate which implications and issues concern this new approach, to outline some ethical challenges regarding the implementation and to present an overview of the ethical arguments mentioned in literature against introducing GDMOs into the environment, and how they relate to Teilhard de Chardin’s perspective.

1. Teilhard de Chardin’s perspective and his ethics for the future

Although Teilhard de Chardin developed his theories long before the actual advent of gene drive technologies, I would argue here that some of his main concepts stand in correlation with the actual arguments already used in the ongoing discussion. Furthermore, I will here argue that his concepts may be helpful in resolving the questions and doubts we are now having in the face of challenges that gene drive use represents for society. Although he was not explicitly an ethicist, his thoughts and concepts and the role of responsibility represented, as Ana Jeličić argues, his *ethics for the future* that are based on the positive picture of the world that needs to be developed if we want to have a future in it (Jeličić 2015). This *ethics for the future* in Teilhard de Chardin’s work found itself in an evolutionary perspective where the Earth is not perceived anymore as an already harmoniously and steadily organised entity where our only task is to preserve it, but rather the Earth is more a *New Earth* to construct (Teilhard de Chardin 1968, Galleni 2016). However, by the construction of this *New Earth*, he does not mean dominion over nature as has been part of our culture for centuries, but it means to construct a future for humanity. His vision of the Earth as a mutual home for all living beings, human and non-human, demands a responsibility towards this home where only the preservation of a mutual home automatically means the survival of humankind too. Furthermore, this reconstruction of the Earth is presented as *moving towards* (forward) the Omega point (Galleni 2014). As matter of fact for Teilhard de Chardin, the progress in itself is not an absolute value, but it is more *moving towards* the future and preservation of life, which entail some responsibilities (Jeličić 2015). This *moving towards* is continuing progress that represents moving towards a complexity, and thanks to this complexity the biosphere^4^ creates the noosphere^5^ (Galleni 2016). The biosphere as a concept has been recently reorganised by J. Lovelock and Lynn Margulis where they mentioned it in the form of *biota* as a part of the *Gaia hypothesis* (Lovelock 1999, Margulis 1999). Lovelock and Margulis claim that different parts of the biosphere interact, as Ludovico Galleni depicts, in the form of retroactive ‘rings’ creating a strong ‘chain’ maintaining the biosphere’s stability. This stability can be achieved, according to Lovelock and Margulis, through diversification and the increase of complexity, in other words, increasing the quantity of the rings within the chain and improving their quality (Galleni 2001). However, this biosphere is not simply a summary of different parts or ecosystems, but it is a new ontological entity. In order adequately to understand this new ontological entity and the evolutionary processes underlying it, according to Ludovico Galleni, Teilhard de Chardin gives priority to the science of biology, which might contribute to its understanding (Galleni 2016). Therefore, *moving towards* not only represents openness for the future, constructed with technological progress, but also the defence of the homeostasis of the existing equilibrium within the biosphere. Defending the actual equilibrium in the biosphere represents a gathering point for all of humanity and an enabling point for the creation of the noosphere where its main aim is to proceed towards the future (Galleni 2016). The realisation of the noosphere for Teilhard de Chardin, as Ana Jeličić points out, not only involves the emergence of a global network of stakeholders but also a more organised global policy, as well as reciprocal interaction with the mutual project (Jeličić 2015). Therefore, this mutual project is an essential precondition for the creation of the noosphere receiving its global character where the noosphere receives the character of, as Hub Zwart emphasises, “a conscious reshaping of the world, an epochal transformation affecting the entire planet” (Zwart 2016b, p. 403). This paradigmatic approach introduced by Teilhard de Chardin changes the existing perspective where the environment and Earth are only at the service of humanity to realise itself, to self-awareness of the preservation of the biosphere’s stability becoming a precondition for humanity to *move towards* the future.

An interesting fact relating to his idea of the *New Earth to construct* is that De Chardin in this process does not implicitly reject the possibility of the use of technology in this Earth reconstruction process, as one of the strong characteristics of his ecological approach, comparing perhaps to environmentalists. In fact, for De Chardin technology is an instrument that might provide his *moving towards* the future of humanity (Galleni 2016). However, we could agree that not all technologies are suitable for inclusion in this construction of the *New Earth* process; furthermore, they need to fulfil three key criteria irreversibility, proportionality, and foreseeability. At the first sight, gene drive technology has the potentiality to irreversibly change the ecosystem, and because of it that could be considered as inherently bad. Nevertheless, this cannot mean that every irreversible change might be considered as inherently bad; therefore, to distinguish them properly Teilhard’s concept of the biosphere’s stability might shed some light of clarification. Furthermore, relying on the Teilhardian assessment that only the irreversible changes that harm the biosphere’s stability might be considered as inherently bad. Proportionality and foreseeability comes after we have acquired adequate knowledge and understanding of the underlying mechanisms of the gene drive technology; however, at the moment this understanding and knowledge is veiled with an inevitable degree of uncertainty.

Furthermore, as Ludovico Galleni points out Teilhard de Chardin has insisted on the great necessity to study the laws of the biosphere and the underlying mechanisms in order to obtain the stability of the biosphere (Galleni 2016). To continue this walk towards the common future there is no need to stop the technological progress, but there is a need to acquire adequate knowledge in respect of underlying processes of biosphere stability and to include the technology in a way which preserves this stability and doesn’t transform it irreversibly.

This mutual project, seen in the symbiosis between the biosphere-noosphere, needs to be based on practical solutions and long-term political aims, and one of these long-term aims might be finding the proper way to address the issue of vector-borne diseases without endangering the stability of the biosphere.

2. Promises and perils of gene drive dissemination strategies for public health purposes

Gene drives are well documented in nature as well, in the form of “selfish genes” or “selfish chromosomes” invading different insect species (Nolan & Crisanti 2017). The mere idea of gene drives is not a new one, and it has its roots in the 1960s (Curtis 1968), where it was introduced and proposed as a theoretical principle for the suppression and control of pest populations. The idea of the “selfish gene” has been envisioned as a control of populations that are particularly harmful, which need to be suppressed or even made extinct (Burt 2003). Although the idea of gene drives has existed for several decades, more recently, with the development of gene editing technologies, scientists have tools to alter almost any genome in almost any sexually reproducing species in order to spread these alterations through subsequent generations. Among the first gene editing tools that were successfully created was zinc finger nuclease (ZFN), but the difficult effective design and high costs reduced its use (Reid and O’Brochta 2016). A second gene editing tool, simpler to design, was the transcription activator-like effector nuclease (TALEN); this tool also represented a much too costly approach (Ma et al. 2016; Urnov et al. 2010). Based on the adaptive immune system that can be found in bacteria, the gene editing tool Clustered Regularly Interspaced Short Palindromic Repeats/CRISPR functions as a pair of “scissors” where it can recognise a particular gene in the genome, cut it with a high level of accuracy, and paste a modified one. Cas9 is used mostly with CRISPR and represents a DNA endo-nuclease providing a certain advantage for CRISPR compared to other gene editing tools such as ZFN and TALEN, because it does not require repeatedly designing and expressing the Cas9 protein. Even though this new gene editing tool is a simple, cheaper, and more precise genome editing tool providing scientists with the realisation of the long-lasting dream of gene drives, some technical hurdles still remain in using the CRISPR gene editing tool in gene drives’ design (Zentner and Wade 2017).

The main role of gene drives is to override normal inheritance laws, increasing the possibility of passing the variant over 50% in such way allowing for genetic alterations to spread in a population much faster than in normal conditions (Lunshof 2015). Gene drives’ primary function in a population is to insert the above mentioned molecular “scissors” along with the gene mutation into the desired part of the DNA, where the “scissors” cut the corresponding gene on the chromosome within the genome and substitute it with the mutated gene. These genetic alterations are inherited by the progeny, including the so-called “scissors” that will continue the alteration process when breeding with others (The Norwegian Biotechnology Advisory Board 2017). Gene drives are genes or genetic elements, which cheat the gene transmission processes making certain genotypes over-represented in the progeny, and increasing the frequency of these genotypes in the progeny reduces the possibility of the occurrence of other genotypes. These new drive systems are primarily aimed at causing populations to collapse, i.e. to go extinct, or to increase the frequency of individuals with genotypes resistant to viruses, e.g. dengue or parasites e.g. Plasmodium (Reid and O’Brochta 2016). The first proof-of-concept study to demonstrate that these gene drives can be introduced in organisms were performed on yeast (Di Carlo et al. 2015) and fruit flies (Gantz and Bier 2015b). The results have shown an enviable success rate of nearly 100% in spread through all offspring. After demonstrating the efficiency of gene drives in simple organisms, the next step was to apply gene drives in some more complex organisms such as mosquitoes *Anopheles stephensi* (Gantz et al. 2015a.) and *Anopheles gambiae* (Hammond et al. 2013), introducing genetic changes with the aim of fighting malaria.

We can say that the primary idea and guiding path in the development of the gene drives were their intended applications for a public health purposes, especially to control vector-borne diseases contributing to the existing global health picture. The already mentioned experiments on mosquitoes, which gave positive results, have provided a certain amount of hope in resolving the problems that represent the spread of these vector viral diseases(Reardon 2016). Therefore, this new technology might help in combating these challenging viral diseases malaria, dengue and recently Zika (Carlson et al. 2016).

Although the primary focus of the gene drive development was to fight against malaria, dengue, chikungunya and lately the Zika virus, this is not its only option application in the domain of public health. Mice are carriers of Lyme bacteria, which are transferred to humans through tick bites; therefore it is necessary to engineer a local mice population in order for them to carry antibodies for the Lyme disease bacteria (Noble et al. 2016). This would be a typical example of expanding gene drive use for public health purposes beyond its application in mosquitoes.

It is important to point out that some species are vectors not only for one disease but for several, such is the case of *Aedes aegypti* mosquitoes*,* who are vectors for diseases such as chikungunya, yellow fever, West Nile, eastern equine encephalitis, and dengue virus (Bhatt et al. 2013, Stein 2015), as well as Zika. Despite the fact that malaria represents primary problem among vector-borne diseases, a promising potential application of the gene-drive, in fighting dengue, would be the case of *Aedes aegypti*, because it is much easier to rear and to experiment compared to *Anopheles*; moreover, the dengue life cycle is not so complicated as in the case of the *Plasmodium* parasite (Marshall and Taylor 2009)*.*

The existing literature on gene-drive modifications in fighting vector-borne diseases offers two approaches or strategies. These two methods represent the main strategies in prevention of the spread of diseases, both containing their advantages and disadvantages.

First is a *population suppression* approach, where its primary aim is to change the proportion of males and females, decreasing the possibility for further reproduction (Hammond et al. 2013). With a gene drive that will propagate, encouraging the production of more males than females and directly impeding the reproduction cycle by reducing the population number the chances for population spread also reduce. In some cases, such as malaria, the reduction of females is directly connected with the transmission of the disease, whereas in the case of *Anopheles gambiae* only females bite humans and thereby transmit the *Plasmodium* parasite. Although it seems very effective, it has its downsides and they are more of the long-term nature where this strategy might even result in the extinction of some species.

We have examples from the past where the eradication of species has brought negative consequences for the ecosystem and the biosphere, such as oyster overfishing in the United States causing algal blooms and thus oxygen depletion that led to lower biodiversity (The Norwegian Biotechnology Advisory Board 2017). The extinction of one species does not guarantee the end of vector-borne diseases, as there are diseases where several species may be a vector. For example, the extinction of *Aedes aegypti* will not terminate the spread of dengue or chikungunya because another mosquito species *Aedes albopictus* is also a vector for those, as well as for Zika; furthermore, in continuation with this thinking it automatically opens the necessity for *Aedes albopictus’s* extinction too. The question that poses itself is where do we draw the line? How many species do we need to render extinct to be free from vector-borne diseases? (Sarkar 2016).

The second approach would be *population replacement*, the primary aim of which is a conversion from transmission-competent to a transmission-impaired organism (Gantz et al. 2015a). Gene drives introduced into organisms disable the viral carrying gene, such as e.g. spreading an anti-malarial gene through procreation even if its effects do not carry any reproductive benefits for the mosquitoes. Although this seems a great idea, it is still limited as only one drive can be introduced in the case of one vector disease and therefore represents a particular problem where a specie transmits several diseases such as in the case of *A. aegypti* or when several species are responsible for the spread of one disease such as malaria (Killeen et al. 2002). Perhaps the best solution would be to create a universal gene that might work even in cases when one specie is a carrier for several diseases or for all carriers of the virus/parasite in common. Therefore, although this approach seems very promising and it has fewer diminishing biodiversity consequences, its realisation might rely on coping with some realistic technical difficulties. It is necessary to point out that parasitic resistance, despite the gene drives, is very hard to obtain; over time, there is a chance that this resistance would slowly decrease thanks to evolutionary resistance (The Norwegian Biotechnology Advisory Board 2017). If it would be applied, it is important to emphasise that this strategy requires a constant release of new gene drives to fight a wide range of diseases carried by the same vector.

The limitations of these two presented strategies are directly connected with the ethical issues and their impact on our ecosystem.

3. De Chardin’s *ethics for the future* in the debate on the release of GDMOs

Despite the potential benefits, the application of gene drives is not without ethical challenges regarding their release and functional use. Actually, the aim of this part would be not to present the ethical issues regarding gene-drive modified organisms through anthropocentric polarisation, but above all to present the ethical issues as issues of our biotic community in which we all live. Most current concerns are of consequentialist nature, and they regard many risks based on uncertainty and lack of adequate knowledge of the long-term effects that the implementation of GDMO’s into the ecosystem might leave. Mostly they focus on the debate how to minimise the risk without hindering the prospects of the research, increasing the likelihood of innovation benefits for humans. However, the ongoing discussion should include other values such as the ecosystem, biosphere stability, and biodiversity and not be reduced to the “One Welfare” approach with public health as the only value of interest (Capps et al. 2015). Nevertheless, the implications of the GDMO release regard the consequences for some modified organisms and the modified organisms as a part of the ecosystem. For example, both strategies above proposed for the control of vector-borne diseases have their own implications on the biotic community, some are particularly harming and some are not. However, it is important to point out that at this moment the possible implications are not well understood and they are based on predictions, because of the lack of scientific knowledge and studies that elaborate further implications. The only way to adequately assess these implications is to gain more knowledge about the interactions between different organisms and their actual role in existing ecosystems, and as Teilhard de Chardin points out the science of biology in collaboration with ecology might help us understand and perhaps foresee how GDMOs will affect the entire biosphere. Only they can help to understand the underlying mechanisms in the biosphere stability, and interactions between the species. One of the first things to do certainly would be to evaluate the proportion of the realistic potential risks and the potential benefits. The calculus of the risks should include foreseeable irreversible ecological effects on human health and well-being. However, the ethical assessment should not be reduced to just risk-benefit analysis; moreover, the ethical implications are present from the research and development process of gene drive technology to the implementation and release of the organisms into the environment. Therefore, leading scientists have recommended taking into consideration the potentiality of the gene drives to alter wild populations and ecosystems and to take robust safeguards and methods of control even in a laboratory testing phase to prevent possible accidental releases (Esvelt 2016; Esvelt et al. 2014). Such ecosystem disruption consists of more severe, complex, and system-level consequences that are hard to model and predict (Lunshof 2015). Therefore, the assessment of the ecosystem implications despite the known facts would now imply a great level of uncertainty.

The *population suppression* strategy would have hard implications for an ecosystem, although we can imagine living without some species (Pugh 2016), and with that kind of thinking, we do not take into consideration their actual role in a biotic community. Teilhard de Chardin’s concept of the biosphere might be useful in answering this, where the extinction of species certainly would leave serious implications on the biosphere’s stability, because it would decrease the interacting relationships between its parts and influence the quality of interactions within the entire ‘chain’ that maintains the stability. The diversity of the existing species empowers the stability of the biosphere, increasing the interacting relationships in its complexity (Galleni 2016). Therefore, without proper understanding of the interactions between the species, as well between the species and the environment in their complexity, the population suppression strategy of using gene drive would result in unknown consequences on the biosphere’s stability. Although it seems that this argument leads to the conclusion that no species should be rendered extinct, it does not, because its focus is a good and informed understanding of the actual existing interactions in order to make a decision that strives towards preservation of the biosphere’s stability. Moreover, as envisioned by Teilhard, the construction of the New Earth is not the construction of an Earth with infringed biosphere stability or biodiversity intangibility. Therefore, the decision-making in the case of the GDMOs would be a purely normative enterprise, because the preservation of the biosphere’s stability represents a value that can be aligned as a norm, within De Chardin’s *ethics for the future*. This argument can perfectly be transferred in a real-life setting, in the concrete case of mosquitoes. Some might argue that the role of mosquitoes is not significant and that they are rather pests than insects useful for the ecosystem; however, every species has its role in the ecosystem. Notwithstanding, mosquitoes are considered to have two important ecological roles: first, they pollinate some types of plants e.g. in Alaska *Aedes* pollinates certain types of orchids (Thien 1969) or in the Netherlands certain types of *Silene* (catchfly) (Larson, Kevan, and Inouye, 2011). Second, they are a food source for a variety of birds, fish, and amphibians, and it is known that certain species of mosquitoes are an essential food resource during avian migration in the Arctic. Some scientists even argue that there might be a 50% reduction in the bird population if there will not be enough mosquitoes for them to eat (Fang 2010); therefore, it is essential to understand the proper role with its all implications of a particular species in the ecosystem (Resnik 2012). At the moment, we do not have adequate studies and understanding of how the release of GDMO’s would affect birds, fish or amphibians or the entire biotic community (Appadurai. et al. 2016). Removing one species and creating a niche might create a place for some other species to take its place, and therewith disturb the biosphere stability. Throughout history, we have had the chance to experience how some species have been brought from another habitat and introduced to a new environment, such as in some regions of South and Central America and the Caribbean where *A. aegypti* have been introduced from Africa. Moreover, some may even argue that the eradication of such introduced species would not contribute to ruining ecosystem balance; rather, it is more a removal of non-domesticated species. However, removal of, e.g. *A. aegypti* that is a carrier of particular pathogen might be counterbalanced and replaced in larger numbers by another mosquito, e.g. *Culex pipiens*. The substitution of a species by another species, such as *Culex pipiens* opens up new possibilities that might lead to new pandemic or endemic situations; in the case of *Culex pipiens* there is a danger of a West Nile virus pandemic (Ciota et al. 2013). Nevertheless, it is not to exclude that these gene-drive modified organisms through their mutation and evolution might become carriers of other pathogens.

Gene drives have been foreseen for a target species, but at the moment it still remains to see if the gene drives will remain in the same species or if they will, through a process called horizontal transfer, jump from one organism to another even in the case of unrelated species. If society decides to use the first strategy *population suppression -* a gene drive that is intended to reduce the number of a population by killing them might jump to other species such as bees and eradicate them, which is a threat to the ecosystem because of their very critical roles in pollinating and honey producing. This phenomenon is called the “hopping gene”, where the dissemination and spread of the gene drive can go to “non-target” species (NAS 2016). This effect is of particular importance for a release of gene-drive modified organisms and it is necessary to understand it more thoroughly, particularly when the main aim is to decrease the organisms’ reproduction power, may even lead to an extinction of a wrong and unintended species. Surely, at this point, other off-target effects of the GDMO release require study and be properly demonstrated. Not only do the horizontal transfer and off-target effects of the GDMOs need to be seen, but also how this change will be accepted by the organism itself, whether it will weaken the organism’s ability to adapt to the changing environment especially in cases when some of these adaptations belong to the natural selection process.

At present scientists have little or no knowledge about how these gene drives will interact with the evolutionary robustness of the organism, and how they will move through the entire population or just through part of the population because of the mutations that may occur (Oye et al. 2014). Therefore, in order to observe this evolutionary robustness, pilot studies in highly controlled and contained glass-houses would be essential (Brown et al. 2014, WHO 2014). Moreover, the evolutionary robustness would be important in order to assess the possible repeated releases of gene drives, especially in cases when the *population replacement strategy* will be applied. However, if the release would happen, it still remains to be seen how and for how long these genome edited organisms will survive in a natural environment (Caplan et al. 2015). Another problem that might arise according to biologists is a species resistance to gene drives, in other words, the fitness will eventually decrease and it will be through evolutionary course eliminated by the fitter genes and the only way to prevent this is to continue releasing new gene drives into a population (Wade 2015, Callaway 2017). Moreover, the release process will turn out to be a closed circle requiring new releases of the updated gene drives and this closed process represents a point of no return. Therefore, the gene drive would in this case leave irreversible changes on biodiversity and it would remain to be seen how it would affect the biosphere’s stability.

Nevertheless, scientists argue that creating another immunising or reversal gene drive that will restore the organism to the previous state is actually a point of return or in other words a way back. Immunising gene drives are intended as a countermeasure in case of unwanted consequences where it might block the spread and propagation of the unwanted gene drives within the organisms, by altering the target sequences. This reversal gene drive will restore the organism to the previous genetic state, and even to the original genetic sequence overwriting the introduced changes inserted by the initial drive (Oye et al., 2014). Although the primary idea of the reversal gene drive is a recovery switch from the inserted genetic changes, it cannot be said that it is a recovery switch for the ecological changes that would be introduced to the ecosystem. Even if this recovery gene drive works, it will still represent a problem in cases of crossbreeding interactions between the modified and non-modified species (Resnik 2012). It is not even sure whether these recovery and reversal genes may help to recover and restore the changes in cross-breed organisms. However, to observe such interactions among the species may only be possible through field release (Oye et al., 2014). The availability of these reversal drives may make people become overconfident and assured that they can really control all the implications of the gene drives, and even if it causes unforeseen consequences they may believe they could correct them. The same two characteristics of human hubris have brought and created many environmental problems, where humans have thought they could control nature (NAS 2016, p. 69). Moreover, the same hubris has constructed in a society a worldview of masters of bio-community and not the stewards of the same. The stewards of the bio-community enter into the much broader concept of “Planetary stewardship” envisioned as a third stage of the Anthropocene where we will need to “develop a strategy to ensure the sustainability of the Earth’s life support system” (Keulartz & Bovenkark, 2016 p. 4).

The National Academy of Sciences through a deliberative and legalised approach, emphasising that scientists need more to engage the affected communities, come to a similar conclusion that “there is no sufficient evidence available at this time to support the release of GDMOs into the environment” (NAS 2016, p. 166, Abbasi 2016). However, their deliberative path is focused only on the uncertainties over science rather than concerns over the biosphere’s stability. This only empowers the necessity and adequacy of the Teilhardian ouvre within the GDMO discussion, where the broader context and values are taken into consideration and not only consequential uncertainty over science. Furthermore, it does not underestimate the consequences and takes them seriously into consideration within the foreseeability process of the potential implications on the biosphere’s stability. In order to prevent such consequences and to reduce the chances of harmful effects on the ecosystem, proper international regulation is needed. Actually, there is a lack of unified guidance for the modification of non-human organisms; at the moment there is only one regarding prevention of research and development of biological weapons, entitled *Biological and Chemical Weapons Convention* (Caplan 2015). There is also a need for international regulation based on careful study and continual ecological monitoring. The international regulation that refers directly to genetically modified organisms is *United Nations Convention on Biological Diversity* and its supplement *Cartagena Protocol*, which addresses the issue of the control of movement of genetically modified organisms such as a prohibition of release of organisms that are not allowed to cross international boundaries. International trade rules may also factor in the release of these gene-drive modified organisms. However, due to the nature of GDMOs, they are likely to spread invasively across national boundaries, and as such we need new and updated international regulations. Furthermore, not all countries have signed the *Cartagena Protocol*, only 168 countries have done so (Resnik 2012). One of the leading countries in genetic modification, the USA, has not signed it (Champer et al. 2016).

In order to set up international regulations, one of the essential preconditions would be to find an agreement whether to release the gene drives or not and when. Why is an international agreement necessary? It is necessary because, as mentioned earlier gene drives are not aware of national borders and they will affect even those that were not engaged in decisions about release. Therefore, every release or even a field release would be a challenge for global society, even in cases when all safety and regulatory measures have been taken. The only way to achieve a global agreement whether to use GDMOs and release them into the environment is only possible in a process when people unite to express their values, attitudes and positions to find a solution for a better future. Only when the question regarding GDMOs becomes a part of the mutual project of humanity, or in Teilhard de Chardin’s words with the creation of the noosphere, will a responsible decision with a common interest be achieved. Therefore, to achieve this responsible decision we can agree it is only possible through a good ecological understanding of the system we are trying to manipulate, meaning also an adequate understanding of biosphere complexity and how the introduction of GDMOs might affect its stability. Starting from the knowledge of the actual situation, society can build foreseeable scenarios as to what may happen if GDMOs are released and whether it would bring irreversible changes to the biosphere’s stability. If the decision to release GDMOs finds its place, then humanity will need - before their release - long-term monitoring plans to be prepared for unforeseen changes enabling their easier detection, and actual backup plans for fixing it if something goes wrong. Furthermore, the challenges of the biosphere’s stability are also a precondition for the creation of the noosphere with a mutual project of finding a global agreement on the release of GDMOs as a part of the moving towards humanity’s future. The global agreement would mean also reaching a consensus about our shared values circulating in the GDMO release debate. Importantly the virtue of humility must be present in the research domain from which the transparent information of possible benefits and harms of GDMO release will be provided to the global public.

## Concluding remarks

Two things come to mind when we speak about this kind of ecological or environmental engineering. First of all we must acknowledge that we are biological creatures living as a part of this biotic community and to find a *modus vivendi* (way of living) with other living organisms, but on the other hand we are creators with a *Promethean* desire that grows and further reflects in our engineering capability facilitating at the same time the same *modus vivendi* in our ecosystem. Therefore, we, human beings as creators and creatures at the same time, should find, as Bruce Jennings says, a realistic balance between these two serving roles that will therewithal reflect in a value of humility (Jennings 2016). Finding the exact balance might be helpful for finding the solution for the GDMO release, where we do not underestimate the world in which we are living and how it functions, and also, so that we do not hesitate from our ability to produce and use technology to make a better future for us.

One of the obligations and responsibilities that society has is to construct a better future as a way of preserving our common home. Although the GDMOs potentially have large benefits for human health, there are potential harms that still need to be assessed and understood regarding possible severity. Nevertheless, at the beginning, the question posed was should we use and release the GDMOs or not? Following Teilhard’s oeuvre, in order to answer this question, we need to have an adequate understanding of all the factors to be able to foresee the consequences and effects of their release. Thereafter, summing up all the factors and effects will show how it will relate to the existing biosphere stability where the stability of the biosphere will need to prevail in the decision-making. In other words, the primary focus should not only be on different parts or perspectives, the public health benefits, or implications for the environment, but the integral picture concentrated on the preservation of the biosphere’s stability as a whole. Observing and making decisions from this integrative picture there is a chance to find a good balance between both public health benefits and environmental implications. In this preservation of the biosphere stability, we should, instead of putting ourselves at the centre of the world as being in this world or in the ecosystem, change the orientation into being with other members of our biotic community, including those that will come after us, i.e. our progeny. In other words, our decision-making should be guided by the principles of co-existence and co-emergence with others or in other words by intergenerational responsibility (Zylinska 2014). This reorientation perhaps would be crucial to receive clarification for doubts that we now are actually having. However, the issues of ecological engineering are not only a matter of scientific or industry assessment of positive and optimistic impacts, but they are also an issue of community engagement through transparent, informed, and fully inclusive public discussions. This reorientation towards others and preserving our mutual home will become a mutual aim for all of humanity as part of the common project, and at the same time become a motivator for the creation of the noosphere in order to construct a *New Earth* for our better common future.

## Footnotes

1 There are many definitions of the term Anthropocene as to what that exactly should be, but following one includes all the aspects: “The term Anthropocene suggests: (i) that the Earth is now moving out of its current geological epoch, called the Holocene and (ii) that human activity is largely responsible for this exit from the Holocene, i.e. that humankind has become a global geological force in its own right.” (Steffen et al. 2011)

2 Vector-borne diseases- Vector-borne diseases are illnesses caused by pathogens and parasites in human populations.

3 Biotic community- all living organisms interdependent and interacting with each other inhabiting the same environment

4 Biosphere - includes all living and non-living beings where their complex relations represent an evolving entity.

5 Noosphere - represents the union of all thinking creatures, i.e. humankind, as Teilhard de Chardin indicates, “the psychically reflexive human surface.” (Teilhard de Chardin, 1999, p. 74.)
